# Magnitude and associated factors of wasting among under five orphans in Dilla town, southern Ethiopia: 2018: a cross-sectional study

**DOI:** 10.1186/s40795-019-0295-6

**Published:** 2019-06-20

**Authors:** Andnet Tadesse Wete, Tadesse Alemu Zerfu, Adane Tesfaye Anbese

**Affiliations:** 1 PATH ETHIOPIA, Dilla, Ethiopia; 2 Department of Public health, School of Public Health, College of Medicine and Health Science, Dilla University, Dilla, Ethiopia

**Keywords:** Wasting, Under five orphans, Dilla town

## Abstract

**Background:**

About 24 million children across the world live without their parents. In resource-limited countries like Ethiopia, childhood malnutrition is common and intertwined with poverty. It is a leading cause of death for children contributing over half of child mortality in sub-Saharan Africa. Nevertheless; little is known about the prevalence of malnutrition and associated factors among under five age orphans, as most of the variable studies were geared towards under five children as wholesome.

**Method:**

A community based cross-sectional study design complemented with qualitative methods was conducted collecting data from mothers/caretakers of 367 orphans in Dilla town, Southern Ethiopia from 5, Dec.2017–18, Jan. 2018. Systematic random sampling technique was used, A structured pretested interviewer administered questionnaire complemented by focus group discussions and key informant in-depth interviews" was used. Anthropometric measurements were also carried. Data were entered in to EPi-info version 3.5.4 software and exported to SPSS version 20 for analysis. The prevalence of wasting among Orphans was assessed by calculating the percentages using ENA for SMART 2012 software and analysis was made using WHO Standard cut off point below- 2 S. D using z-scores. All variables with *p* value of < 0.25 during bivariate logistic regression analysis were entered to a multivariate analysis to identify variables independently associated with the outcome variable at p value 0.05 with 95% CI. For qualitative aspect, thematic framework analysis was employed.

**Results:**

11.1% orphans were wasted from which 3.3 are severely wasted. The main associated factors of wasting were found to be number of under-five orphans at home (AOR 1.420; 95% CI 1.094–3.086), duration of breast feeding (AOR 2.039; 95% CI 1.027–4.048), marital status of care givers (AOR 1.482; 95% CI 1.692–3.377), age when complementary meal started (AOR 2.023; 95% CI 1.028–3.980), wealth index (AOR 2.558; 95% CI 1.074–3.515) and access to balanced diet (AOR 2.022; 95% CI 1.026–3.889)

**Conclusion:**

The prevalence of wasting is high among under-five orphans; therefore, all concerned bodies should pay a great attention for proposed interventions like Strengthen the social interactions and indigenous institutions to maximize social care for under five orphans and Integrating locally available nutrition support programs to reach under 5 yrs orphans.

**Electronic supplementary material:**

The online version of this article (10.1186/s40795-019-0295-6) contains supplementary material, which is available to authorized users.

## Background

Wasting represents an acute form of undernutrition, and children who suffer from it face a markedly increased risk of death, increased risk of diseases and low productivity [1].

Most of the available evidences are related to the general community of under-five children which have differences of risks and predisposing factors as well as severity levels. It is believed that about 24 million children across the world live without their parents, and numbers of children live outside parental care; but widely neglected to get attention in studies [2, 3].

Due to the impact of AIDS, conflicts and other many more reasons, millions of children have been orphaned in the African continent and an estimated 12.3 million children have been orphaned in sub-Saharan Africa [4–6]. This orphan population will increase in the next decade as HIV-positive parents become ill and die from AIDS and vulnerability also keep increasing because of associated factors [2, 4]. In Ethiopia close to 5.5 million children (6% of the total population) are categorized as orphans and vulnerable children (OVC) [7]. The rate of undernutrition among under-five children in Ethiopia is among the highest in the world and Sub-Saharan Africa, 9% of under-five children were wasted. It is the major cause of illness and death among under-five children in the country [8, 9].

A number of factors have been suggested to affect both the level of food security at household level and the children’s nutritional status, some of which are independently associated with households in which orphans and vulnerable children live. These can broadly be classified into child characteristics (e.g., age and gender), household characteristics (e.g., wealth index, and number of children in the household), parental characteristics (e.g., occupation, education level and age of the household head) and community factors (e.g., water supply and hygienic practice) [9–12]. Because of the severity of related problems, nutrition is placed at the heart of Sustainable Development Goals’ (SDG) and vital for achieving 12 out of 17 SDGs [13],

Generally, Gedeo Zone; lacks information concerning nutritional status of under-five orphans, while children 6 to 59 month in this segment of population are potentially at greater risk of undernutrition due to poor nutrition, less social and medical care. Therefore, this study estimated the magnitude of wasting and identified the potential factors affecting Wasting of under-five orphans; specifically wasting in Dilla town, Gedeo Zone, SNNPR, Ethiopia.

The aim of the study was to assess the nutritional status and associated factors of 6–59 month orphans in Dilla town, Southern Ethiopia, 2018.

## Methods

Community based cross-sectional study design complemented with qualitative methods (Focus Group Discussion/FGD and Key Informant Interview/KII) was used. The study was conducted in Dilla town, Gedeo zone, Southern Ethiopia from 5, Dec.2017–18, Jan.2018. Dilla town is located 359 km from Addis Ababa (the capital city of Ethiopia, and the main road from Addiss Abeba to Nairob Kenya crosses the center of the town. Its astronomical location is 6°20′ North Latitude and 38°13′ East Longitude. It comprises nine kebeles (the lowest administrative unit in Ethiopia) The total population is 94,189 out of which 46,058 (49.9%) are males and 48,131 (50.1%) are females. Though the total number of orphans in Dilla town was not known before, according to complete enumeration carried out for the sake of this study, it is 2895.

### Source and study population

The source populations were all orphans aged 6–59 month with their caregivers who were considered for the quantitative and anthropometric survey; mothers, elders, experts from town gender office and community leaders were considered for the qualitative aspect. Whereas Study population was randomly selected orphans aged 6–59 month with their caregivers in selected kebeles of Dilla town.

#### Inclusion criteria

Orphans aged 6–59 month in selected kebeles and which were identified to be included in sample.

#### Exclusion criteria

Orphans 6–59 month of age that are known severely ill because of chronic diseases, like Human immunodeficiency virus/ HIV/ Acquired Immuno Deficiency Syndrome/ AIDS.

and those with immobility precautions like fracture.

### Sample size determination

The sample size of Orphans included in the study was calculated using the formula for single population proportion, just based on the prevalence rate of Wasting (45.7%) from study conducted in Gonder city, Northern Ethiopia, 2014. Considering 95% confidence interval and 5% marginal error; n = $\frac{z^2\ast p\ast \left(1-p\right)}{d^{2.}}$

=381 with 10% non-response rate = 381*0.1 = 420

Since the total population is < 10,000 correction was made and final sample was 367

### Sampling technique and procedures

First, all kebeles in Dilla town were identified by name and complete enumeration has been conducted by preparing “complete enumeration format” for the sake of this study and all under five orphans in Dilla town have been identified; and then by using simple random sampling technique three kebeles were selected and the sample size for each kebele has been proportionally allocated. So, the sampling frame has been prepared based on complete enumeration format register and subjects for the household survey have been identified. Snow ball and judgmental sampling technique was used to involve all possible cases from mothers, elders, experts from town gender office and community leaders just purposively to obtain information through 2 FGD and key informants For qualitative aspect.

Study variables are Wasting, Demographic factors (age, sex, education, number of children in HH, marital status), Socio economic variables (wealth index, employment), Child health care (immunization, sickness), Environmental /sanitation factors (source of water, domestic waste disposal), Food insecurity, Dietary intake (child feeding and practices).

### Data collection instruments and procedure

A pre-tested structured interviewer administered questionnaire was used for data collection in one of the kebele out of selected for sampling; it was adapted from different relevant studies and standards to meet the purpose (8,12,13). Initially, the questionnaire was prepared in English and translated into Amharic and Gedeaufa to obtain information on demographic characteristics, socio economic status, sanitation and hygienic conditions, feeding practices and child care, HH food security status, immunization status, exposure to diarrhea and acute febrile illness of the under 5 years orphans.

### Quality assurance

The data collection was facilitated by 2 volunteer HDA guiders at each kebele who know the house with selected orphans’ well. The data was collected by 6 data collectors; who were diploma nurses/urban health extension workers and managed by 2 supervising health officers. Prior to the commencement, data collectors and supervisors were given 2 days refreshment training by PI in Haroresa health center on the objectives of the study, on the contents of the questionnaire, on the methodology of the study, inclusion and exclusion criteria, on the issues of the confidentiality of the responses, on the use of instruments, on the procedures how to take anthropometric measurement by Nurses and reduction of error: for the first day and then re-demonstration of anthropometric measurement and pretest training for the next day. All measurements were carried out using standard procedures by explaining the procedure to the mothers, fathers, or caregivers. The data collection, application of standard procedure and accuracy of test results were supervised and checked daily for its completeness and consistency through close follow up of PI. For anthropometric measurements, average result has been considered. Then after, the collected data was back translated into English to ensure quality.

#### Anthropometric measurements

Age was collected from mothers, fathers or caregivers; and then was cross-checked with birth certificate; Sex was recorded as male or female; Weight of the child was measured to the nearest 0.1 kg using 25 kg hanging spring scale; in light cloths and without shoes; MUAC: was measured using color coded standard MUAC tape meter by calculating the midpoint of the child’s left upper arm by first locating the tip of the child’s shoulder and the tip of the elbow through right angle position and measurement was taken in the midpoint by straighten the child’s arm and read the measurement to the nearest 0.1 cm. Anthropometric measurements were taken two times and averages taken.

#### For qualitative aspect

The data was collected from 2 FGD, each has 8 members that comprise: mothers/caretakers, elders, town gender office experts and community leaders; and also some key relevant informants.

Operational definition and definition of terms

- **Nutritional status assessment**: was an assessment carried out to understand the magnitude of undernutirition in under 5 years orphans at Dilla town.
- **An orphan**: a child aged 6 to 59 month, living in Dilla town and whose mother, father, or both have died or can be referred as maternal, paternal or dual orphan respectively.
  Standard Definition
- **Wasting**: is weight-for-height/length below minus two standard deviations (<−2SD) from the median of WHO reference population.
- **Diversified balanced diet:** Food intake that includes all of the diversified dietary needs of the organism in the correct proportion**;** the access would be measured based on the cutoff point during analysis: households with poor access, those scored 1 and households with good access, those scored 2.
- **Food insecurity:** Lack of adequate physical, social or economic access to food; the household food security status would be measured based on the cutoff point during analysis: food secured households, those who scored 1 and food insecured households, those who scored 2.
- **Wealth index:** Households with poor wealth index are those who scored 1; households with medium wealth index are those who scored 2 & households with rich wealth index are those who scored 3.

#### Data processing and analysis

Data was entered and cleaned up using EPI-info version 3.5.4 and ENA for SMART 2012 version software was used for anthropometric data management. Principal component analysis (PCA) was carried out for the reduction of variables involved in wealth index, food security status and balanced diversified diet assessment.

Principal component analysis (PCA) was carried out for the reduction of variables involved in wealth index, food security status and balanced diversified diet assessment and factor one would be considered to address extracted components:

Fifteen variables (Electricity, Watch, Radio, TV, Mobile, Refrigerator, Separate room used for kitchen, Bicycle, Any land used for agriculture, Livestock, Account in bank or credit association, Cement/ceramic floor, Corrugated iron/cement/concrete roof, Mud/wood with mud & cement wall & Pit latrine) would be entered into the pool of analysis for the assessment of wealth index and the rank would be assigned into three categories from lowest to highest values; so that 1 was given for poor households, 2 for medium households & 3 for the consideration of rich households. The variables had been entered into the pool of PCA, whereas only 4 components whose load Eigen value greater than one were taken under the first factor and categorized as wealth index status:

Nine variables (Worry about not having enough food, Not eat preferred food, Eat just a few food, Eat food preferred not to eat, Eat smaller due to food lack, Eat fewer due to food lack, No food at all, Sleep hungry & Whole day eat nothing) would be entered into the pool of analysis for the assessment of household (HH) food security status and the rank would be assigned into two categories from highest to lowest values; so that 1 was given for food secured households & 2 for the consideration of food insecure households.

12 variables (Cereals, Roots and tubers, Vegetables, Fruits, Meat, Eggs, Poultry, Pulses and nuts, Milk and milk product, Oils and fats, Sugar, honey/soft drinks, Spices, condiments, coffee, tea) would be entered into the pool of analysis, based on the seven diversified food groups for the assessment of access to balanced diversified diet and the rank would be assigned into two categories from lowest to highest values; so that 1 was given for poor access & 2 for the consideration of good access.

## 24 HRS dietary recalls has been administered by locally assigned diploma holder nurses’ using the multiple pass 24HR technique and the result was compiled by factor analysis

Logistic regression was the mode of analysis and further the data was cleaned and analyzed using SPSS Windows version 20. After having all variables with *p* value < 0.25 as candidate during bivariate, multivariate analysis was carried out to see the effect of each independent variable on nutritional status explained as wasting at *p* value 0.05 with 95% CI. Finally Bar graph was used for diagrammatic summarization of categorical variables and tables were used for summarization variables. **For qualitative aspect**: the data was recoded and typed again in a way suitable for thematic analysis; finally the result was incorporated with the findings of quantitative aspect for sound understanding. **Steps followed during qualitative data analysis**:-Familiarity developed with the data -Initial codes generated - Search for themes/recurring ideas or the subject of a talk- Review of themes - Themes defined and named -The report produced.

### Ethical consideration

Ethical approval and clearance was obtained from the Institution Review Board (IRB) of Dilla University College of health science and Referral Hospital. A mother or father or care giver of each study participant was informed about the research objectives, methods and techniques in detail and informed consent was taken. And then the consent was ensured and confidentiality was maintained. As a value adding consideration, ‘nutrition education’ was given to study participants.

## Results

### Socio-demographic characteristics

A total of 361 aged orphans 6–59 months were participated in the study making response rate of 98.4%. The non-response rate was due to refusal to participate in the study. Regarding sex, 189(52.4%) of the study participants were females and 172 (47.6%) were males. Households who have two or more orphan child are 69(19.1%) and 292(80.9%) have one orphan child. For all Socio-demographic information (Table 1).

**Table 1:** Socio demographic characteristics of study participants, in Dilla town, Southern Ethiopia, 2018

Variables	Category	Frequency	Percent (%)
Age of a child^1^	6–11	1	.3
	12–23	17	4.7
	24–35	96	26.6
	36–47	87	24.1
	48–59	160	44.3
Sex of a child	Male	172	47.6
	Female	189	52.4
Under five children in HH	< 2	292	80.9
	> = 2	69	19.1
Family size	< 5	155	42.9
	> = 5	206	57.1
Orphans’ status	Double orphans	67	18.6
	Maternal orphans	202	56.0
	Paternal orphans	92	25.5
Respondent’s relation with a child	Mother	89	24.7
	Fathers	79	21.9
	Sister/brother	59	16.3
	Grand parent	127	35.2
	Other relative *	7	1.9
Sex of a care giver	Male	19	5.3
	Female	342	94.7
Age of a caregiver	18–49	302	83.7
	> = 50	59	16.3
Marital status of a caregiver	Single	63	17.5
	Married	298	82.5
Head of a family	Mother of the HH	112	31.0
	Father of the HH	233	64.5
	Other **	16	4.5
Education status of a caregiver	No formal education	140	38.8
	Grade 1–8	106	29.4
	Grade 9–12	76	21.1
	Above grade 12	39	10.8
Occupational status of a caregiver	Unemployed	171	47.4
	Employed	190	52.6

^1^in month

*uncles, aunts

**First born, uncles, aunts, in-laws

### Feeding practice and dietary intake

343(95%) orphans had at least once and only 92(25.5%) were known that they were exclusively breast fed, but more than half, 247(68.4%) were not known whether they were exclusively breast fed or not. Above 50% of orphans were breast fed for more than 6 month and 18(5.0%) not fed at all. And 152(42.1%) orphans were started complementary feeding within the age range of 1–6 month, 167(46.3%) and 24(6.6%) orphans were started complementary feeding within 6–12 and > =12 months respectively. Porridge was the first complementary meal for 223(61.8%) of orphans followed by milk for 94(26.0%) of orphans. Cup and spoon were used as means of child feeding for 210(58.2%) of orphans and followed by bottle for 91(25.2%) of orphans **(**Table 2**)**.

**Table 2:** Feeding practice and Dietary intake characteristics of the study participants, Dilla town 2018, Southern Ethiopia

Variables	Categories	Frequency	Percent (%)
Ever breast fed	No	18	5.0
	Yes	343	95.0
Exclusively breast fed	No	22	6.1
	Yes	92	25.1
	Don’t know	247	68.4
Duration breast fed in month	Not fed at all	18	5.0
	<=6 month	161	44.6
	> 6 month	182	50.4
Age started complementary feeding	Immediately after birth	18	5.0
	Within 1–6 month	152	42.1
	Within 6–12 month	167	46.3
	12 month later	24	6.6
First complementary food a child received	Milk	94	26.0
	Adult food	44	12.2
	Porridge	223	61.8
Means of child feeding	Hand	60	16.6
	Cup and spoon	210	58.2
	Bottle	91	25.4

### Child health care characteristics

More than 80% of the orphans received a sort of vaccination; however the cross check for vaccination card revealed that only 120(33.2%) of the orphans had completed the recommended vaccine. Majorities, (84.5%) of the orphans received vitamin A supplementation **(**Table 3**).**

**Table 3:** Health related characteristics of study participants, in Dilla town, Southern Ethiopia, 2018

Variables	Categories	Frequency	Percent (%)
Child received vaccine	No	50	13.9
	Yes	291	80.6
	Don’t know	20	5.5
Have vaccination card	No	72	19.9
	Yes	289	80.1
Vaccination status	Not achieved	241	66.8
	Achieved	120	33.2
Vitamin A supplementation	No	56	15.5
	Yes	305	84.5
Measles infection in two weeks	No	338	93.6
	Yes	23	6.4
Fever in two weeks	No	256	70.9
	Yes	105	29.1
Cough in two weeks	No	275	76.2
	Yes	86	23.8
Diarrhea in two weeks	No	293	81.2
	Yes	68	18.8
Bilateral pitting oedema on physical examination	0	354	98.1
	+	4	1.1
	++	2	.6
	+++	1	.3

**Fig. 1:**
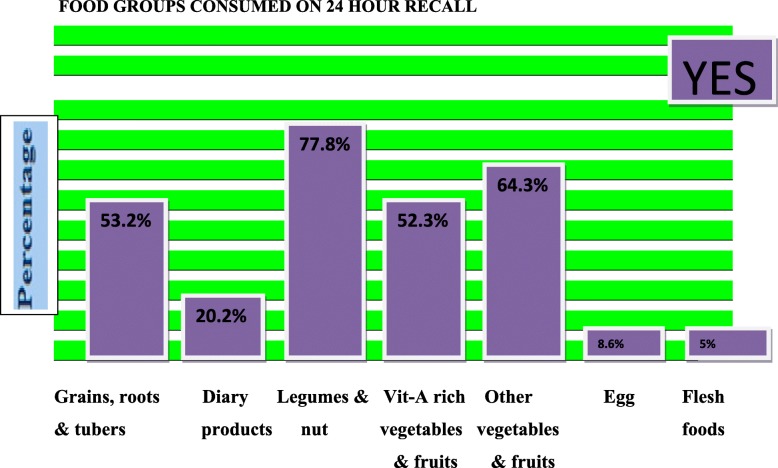
Types of food groups consumed by orphans aged 6–59 months in Dilla town, Southern Ethiopia, 2018

Types of food groups consumed (Fig. 1)

### Factors associated with wasting

The multivariate logistic regression analysis identified number of under five children in the household, marital status of the caregivers, duration that an under 5 years orphan fed breast, age that the child started complementary food, wealth index of the caregivers and access to balanced diet as the determinant factors for wasting.

According to the result of this study, the odds of wasting for under 5 years orphans living within the household two or more under 5 years children dwelling is 1.420 times higher than under five years orphans living within the household which only one under five child dwelling (AOR 1.420; 95% CI 1.094–3.086).

The odds of wasting for under 5 years orphans whose caregivers were single are 1.482 times higher than under 5 years orphans whose caregivers were married (AOR 1.482; 95% CI 1.692–3.377). The odds of wasting for under 5 years orphans who breastfed less than 1 month is 2.039 times higher than under 5 years who breastfed for more than 6 month (AOR 2.039; 95% CI 1.027–4.048).

The odds of wasting for under 5 years orphans who started complementary food before the age of 6 month is 2.023 times higher than under 5 years orphans who started complementary food after the age of 6 month (AOR 2.023; 95% CI 1.028–3.980).

The odds of under 5 years orphans whose caregivers were in poor wealth index category is 2.558 times higher than under 5 years orphans whose caregivers were in rich wealth index category (AOR 2.558; 95% CI 1.074–3.515). The odds of wasting for under 5 years orphans who had poor access to balanced diet is 2.022 times higher than under 5 years orphans who had good access to balanced diet (AOR 2.022; 95% CI 1.026–3.889) **(**Table 4), (Additional file 1)

**Table 4:** Logistic regression analysis showing associated factors for wasting among studied orphans aged 6–59 month in Dilla town, SNNPR Ethiopia, 2018

Variable	wasting		COR (95% CI)	AOR (95% CI)
	Yes (n)	No (n)		
U5 children in HH				
< 2	37(12.67%)	255 (87.33%)	1	1
> =2	11 (15.94%)	58 (84.06%)	1.221 (1.351–3.294)*	1.420 (1.094–3.086)*
Marital status				
Single	10 (15.87%)	53 (84.13%)	1.593 (1.274–3.286) **	1.482 (1.692–3.377)*
Married	30 (10.08%)	268 (89.92%)	1	1
Breastfed duration				
0 month	3 (16.67%)	15 (83.33%)	1.888 (1.020–3.496)*	2.039 (1.027–4.048)*
1–6 month	23 (14.29%)	138 (85.71%)	1.102 (1.00–3.009)* 1.00 (0.380–1.170)	
> 6 month	14 (7.69%)	168 (92.31%)	1	1
Age complementary food started				
Before 6 month	25 (14.71%)	145 (85.29%)	1.873 (1.022–3.432)*	2.023 (1.028–3.980)*
After 6 month	15 (7.85%)	176 (92.15%)	1	1
Diarrhea in two weeks				
Yes	11 (16.18%)	57 (83.82%)	1	1
No	32 (10.92%)	261 (89.08%)	1.078 (0.477–2.479)*	0.920 (0.403–2.096)
Wealth index				
Poor	19 (15.45%)	104 (84.55)	1.980 (0.878–3.868)*	2.558 (1.074–3.515) **
Medium	13 (11.02%)	105 (88.98%)	1.290 (0.922–2.805)*	1.733 (0.691–4.350)
Rich	8 (6.67%)	112 (93.33%)	1	1
Access to diversified balanced diet				
Poor	24 (13.41%)	155 (86.59%)	1.525 (0.839–2.773)*	2.022 (1.026–3.889)*
Good	16 (8.79%)	166 (91.21%)	1	1

*P-value < 0.25 in the bivariate analysis ** *P*-value < = 0.05 in the multivariate analysis

N.B -Vegetables, roots & tubers, fruits, oils & fats, milk & milk product were variables extracted during access to diversified balanced diet analysis.

-Account in bank/credit association, cement/ceramic floor, mobile & separate rooms used for kitchen were variables extracted during wealth index analysis.

## Qualitative findings

### About the magnitude of undernutrition

Both the participants of the FGDs and the key informants revealed that there are a number of orphans and prone to nutritional problems in their community though they are not quite sure of the exact number of these children.

“Amazingly, the number of orphans in community is keeping on the rise just time to time due to HIV/AIDS related death of either parents/both in our town; and the fate of taking care of them is totally left to their poor grandparents and most of them are prone to early death secondary to nutrition related problems.”(KII: from town gender office).

“There are so many orphans in Dilla town; the causes for the death of their parents can be accidents, HIV/AIDS, and other diseases conditions. They are number one to be affected by lack of food and exposed for undernutrition; as a result can’t grow well even physically.” (FGD members)

Generally, participants in both groups pointed out that there are many under five orphans in town; and HIV/AIDS mentioned as leading cause followed by different sorts of accidents and other disease conditions.

### About the associated factors with undernutrition

Community understands under five orphans are more prone to nutrition related problems because of the following reasons stated under their direct speech:

“Formerly, the community members were very much concerned about the care of orphans and deceased family as whole; but now day’s people become selfish due to modernization, the social bond and indigenous institutions are keep weakening; as a result specifically the care for orphans and deceased family as whole is reduced at community level.”(FGD members).

According to one of the KII, the other factor is lack of income:

“The presence of the father/mother by itself is not a guarantee for an orphan child; income is the backbone to solve problems related to nutrition.”(KII: one of the elderly)

“The attitude of the community is quite negative about giving children for adoption in our community; due to that a number of orphans suffering from nutritional problems. Because the community consider as selling them for economic purpose; but the intention is for the sake of themselves. As it’s known, the death of mother or father is beyond the control of a family; so the community or our government has to understand this and giving orphans for adoption should be considered as positive effort for the general wellbeing of orphans.”(KII: one of the mothers)

As stated by the respondents, the depth of under 5 years orphans problem is not limited to undernutrition; but can be forwarded as poor hygiene, lack or shortage of proper clothing, essential social services (such as health, and shelter) in community.

“Though the social bond is weak, community members are trying their best through foster care; so we like to propose the following points as the part of the solution: Specific nutritional support for needy orphans from the government or organizations like university has to be facilitated, evidence based free health service for orphans has to be given, skill training for guardians/family members concerning income generating activities (IGA) with financial support also can be one of the means to solve under five orphans’ nutritional problems in community”(FGD & KII participants)

They believe that, the general wellbeing of orphans will be maintained based on points mentioned above.

## Discussion

This community based cross sectional study complemented with qualitative data, investigated the magnitude of wasting and associated factors among under 5 years orphans in Dilla town, southern Ethiopia, 2018.

As recommended by WHO, evaluation of wasting in this study is based on the reference population of well-nourished children. The weight-for-height is expressed as standard deviation units from the median for the reference group. Children who fall below minus two standard deviations (− 2 SD) from the median of the reference population were referred as wasted.

### Wasting among under five orphans in Dilla town

In this study, the prevalence of wasting is a bit higher than the study findings in Turkey (10.1%), India (9.9%) and Kenya (10%) regional prevalence of Amhara (9.8), SNNPR (6%), Dire dawa (9.7%) and Addis Abeba (3.5%) respectively [14–17]. This might be due to the difference in study segment, study period, socioeconomic characteristics, health service delivery, and study area. Moreover, these studies are on general population,not on orphans. However, the magnitude of wasting in the present study was found to be a bit lower than studies conducted among similar age groups in south Asia (23.4%), Sub-Saharan Africa (22.9%), and regional prevalence in Somali (22.7%), Afar (17.7%), Gambela (14.1%) and Benishangul Gumuz (11.5%), [17]. The variation might be due to involvement of special segments of the study subject who are on care and support.

The prevalence of wasting was found to be consistent with the regional prevalence of Tigray (11.1%), Oromia (10.6%) and Harrar (10.7%), [17–19] respectively. This might be due to similarities in socio economic characteristics and age categories.

The analysis of the study identified number of under five children in the household, marital status of the caregivers, duration that an under 5 years orphan fed breast, age that the child started complementary food, wealth index of the caregivers and access to balanced diet as the determinant factors for wasting.

It’s known that when the same age group available in a certain household, specifically children; everything has to be shared among them to keep stability.

The odds of wasting for under 5 years orphans who are living with two or more under 5 years children dwelling is 1.4 times higher than under 5 years orphans living with only one under five child dwelling. Again, a study conducted on health and nutritional outcomes of orphans and vulnerable children in India showed greater risk of wasting for households have two or more OVC than households have one OVC. This implies that not only the quality, but the quantity of meal is also one of the concerns [14].

Regarding the marital status of the caregivers, The Odds of wasting for under 5 years orphans whose caregivers were single are 1.5 times higher than under 5 years orphans whose caregivers were married. This could be due to the reason that married caretakers have an opportunities to have economic strengthen and other support from their partners than single caretakers and will have an impact on nutritional status of under 5 years orphan so far.

The Odds of wasting for under 5 years orphans who breastfed less than 1 month is 2. times higher than under 5 years who breastfed for more than 6 month. This might be due to the nutritional value of breast milk. This implies that, maternal orphans are at greater risk of undernutrition.

The odds of wasting for under 5 years orphans who started complementary food before the age of 6 month is 2.023 times higher than under 5 years orphans who started complementary food after the age of six month. This might be due to physiologic strength of young orphans to tolerate complementary food before age of 6 month and end up with infection due to the system incompetence.

The odds of under 5 years orphans whose caregivers were in poor wealth index category is 2.5 times higher than under 5 years orphans whose caregivers were in rich wealth index category. The finding of EDHS 2016 and a study held on prevalence of under 5 years malnutrition in Kenya, showed similar result on significant negative association of wealth index and the occurrences of wasting. This might be due to the concomitant effect of low family income and the inflation which further compromised the purchasing power of the household and end up with undernutrition of orphans [15, 17].

The risk of wasting for under 5 years orphans who had poor access to balanced diet is 2.022 times higher than under 5 years orphans who had good access to balanced diet; it implies that, income is one of the concerns.

## Strength and limitations

The study directly addressed under 5 year’s orphans to assess their nutritional problems at grass root community level and Repeatedly counted orphans in two kebeles checked and deleted from the register. Moreover the study used triangulation method to clarify the understanding of association between the variables, these are the strength of this study ·Limitations are related to nature of study design and Hygiene related factors need observation but not done in this study, moreover biases in the data collection is the other limitation.

## Conclusion and recommendations

The study revealed that, the prevalence of wasting is high among under five orphans in Dilla town.

This quantitative study with the complement of qualitative study jointly identified that certain associated factors like Number of under five children in the household, marital status of the caregivers, duration that an under 5 years orphan fed breast, age that the child started complementary food, wealth index of the caregivers and access to balanced diet were associated with wasting

## Recommendations

Based on the above finding of the study, the following recommendations were made:

For zonal health department and town health office:

1. The under 5 years orphans nutrition issues would better be central in all nutrition programs planning to save future generation and requires great attention to be paid at all level, kebeles to zone.
2. Community based nutrition program targeting under five Orphans would be established to tackle the problems of wasting and other health related challenges at community level.
3. Integrating locally available nutrition support programs to reach under 5 years orphans is mandatory to improve their nutritional status.

For local government:

1. Efforts should be made by regional/local government to provide skill training and startup capital for the poor parents/guardians so that they will be economically capable to fulfill the basic needs for under five orphans in community.
2. Intervention programs would also give attention to strengthen the social interactions and indigenous institutions to maximize social care for under five orphans.
3. The local government would strengthen strategies to promote the adoption of under five orphans through legal means to address their nutritional need.

For potential researchers:

Further study would be done to come up with potentially better intervention strategies to address nutritional problems of under-five orphans

## Additional file

Additional file 1: Orphan data. Full data of nutritional status of Orphan and Vulnerable children (CSV 13 kb)

## Abbreviations

AIDS: Acquired Immuno Deficiency Syndrome
AOR: Adjusted Odds Ratio
CI: Confidence Interval
EDHS: Ethiopia Demographic and Health Survey
ENA, SMART: Emergency Nutritional Assessment for Standardized Monitoring & Assessment of Relief & Transition
EPHI: Ethiopian Public Health Institute
FGD: Focus Group Discussion
HDA: Health Development Army
HH: House Hold
HIV: Human immunodeficiency virus
IFPRI: International Food Policy Research Institute
KII: Key Informant Interview
MDG: Millennium Development Goal
MPH/RH: Masters of Public Health in Reproductive Health
NGO: Non Governmental Organization
PCA: Principal Component Analysis
SD: Standard Deviation
SDG: Sustainable Development Goal
SNNPRS: Southern Nation, Nationalities and Peoples Regional State
SPSS: Statistical Package for Social Sciences
UNICEF: United Nations Children’s Fund
WFP: World Food Program
WHO: World Health Organization
